# Exploring the Role of Superplasticizers in Tailoring the Aqueous Dispersions of Graphene Nanoplatelets

**DOI:** 10.3390/ma19143144

**Published:** 2026-07-22

**Authors:** Maria-Evangelia Stogia, George Maistros, Philippe Poulin, Nikolaos D. Alexopoulos

**Affiliations:** 1 Research Unit of Advanced Materials, Department of Financial and Management Engineering, University of the Aegean, 82132 Chios, Greece; mstogia@aegean.gr; 2 ADVISE, 17 Gymnasiarchou Madia St., 82132 Chios, Greece; gmaistros@advise-deta.com; 3 Centre de Recherche Paul Pascal—CNRS, Université de Bordeaux, Avenue Schweitzer, F-33600 Pessac, France; philippe.poulin@u-bordeaux.fr

**Keywords:** polycarboxylate superplasticizer, graphene nanoplatelets, agglomerates, electrochemical impedance spectroscopy, Bode and Nyquist plots, equivalent circuit model, Randles model

## Abstract

**Highlights:**

- Without superplasticizers, higher energy input is required to achieve homogeneous dispersion of high-concentration GnPs.
- Ionic conductivity increases with superplasticizer concentration.
- Superplasticizer concentration significantly affects the required ultrasonic energy.
- Excessive interparticle separation at a high superplasticizer content reduces electrical connectivity between GnPs.

**Abstract:**

Graphene nanoplatelets (GnPs) exhibit exceptional properties for advanced functional applications; nevertheless, their effective utilization is critically limited by agglomeration and poor dispersion. The incorporation of polycarboxylate-based superplasticizers (SPs) enables improved dispersion yet simultaneously introduces insulating effects that hinder conductive network formation. In the present article, we systematically investigate the interplay between GnPs and an SP under varying ultrasonic energy inputs to optimize dispersion and electrical performance through low-cost suspension processing. Dielectric measurements identify the key parameters governing conductive network formation and reveal the dual role of the SP as both dispersant and electrical barrier. Electrochemical impedance spectroscopy, combined with optical microscopy, provides further insights into the state of dispersion and charge-transport behaviour of the suspensions. For the first time, a wide range of SP and GnP concentrations were systematically analysed in terms of electrical properties. The proposed methodology provides a robust and facile approach for on-site characterization of aqueous suspensions with varying GnPs and SP concentrations. Furthermore, an equivalent circuit model is developed to quantitatively validate the experimental results, offering deeper insights into the underlying conduction mechanisms. GnP concentrations of 0.15, 0.50 and 1.00 wt.% were investigated at varying ratios of SP to GnP (0, 1, 2, 4, and 8). Dispersions without SP addition require ultrasonication up to 80 kJ for the GnP agglomerates to break. SP addition at a quantity equal to GnPs (SP1) reduces the amount of appropriate ultrasonic energy for creating a conductive network up to 65 kJ and even more (SP2) at 45 kJ. The fourfold (SP4) and eightfold (SP8) ratio of SP to GnP require higher ultrasonic energy, up to 82 kJ and 70 kJ, accordingly.

## 1. Introduction

Scientific interest in smart materials has shifted toward optimizing the parameters that enable their functionality [1]. Since nanomaterials are often dispersed for incorporation into building materials for sensing, understanding their electrical transport mechanisms is crucial to achieve stability and optimal properties [2]. The aggregation kinetics of colloidal systems are highly complex and difficult to control due to multiple influencing factors, including particle concentration and composition, system polydispersity, solvent characteristics, temperature, pH, and ionic strength [3]. Different techniques will be analysed in the following text.

Kauppi et al. studied dispersion stability by colloidal probe atomic force microscope (AFM), measuring van der Waals, electrostatic, steric, and solvation forces between particles in liquid [4]. Dynamic light scattering (DLS) provides nanoparticle hydrodynamic size and polydispersity (PDI > 0.3 indicates non-uniformity), while depolarized DLS probes rotational diffusion, suitable for anisotropic particles like graphene nanoplatelets (GnPs) [5]. In another research study, DLS measurements revealed that GnPs aggregate rapidly even at low ionic strength, indicating weak electrostatic stabilization and dominant van der Waals interactions [6]. Electrophoretic light scattering (ELS) measures zeta potential (ζ), where |ζ| > 30 mV indicates good electrostatic stabilization, while ionic strength affects stability by altering double-layer repulsion [7].

Another widespread analytical technique is Fourier Transform Infrared Spectroscopy (FTIR) that measures the absorption of infrared light at frequencies corresponding to bond vibrations, enabling the identification of surface functional groups (e.g., –OH, –COOH) and chemical modifications. An analysis of polycarboxylate superplasticizer (PCE) FTIR spectra revealed characteristic peaks at 1729 cm^−1^, corresponding to C=O stretching in the acrylic acid backbone, and at 1123 cm^−1^, assigned to C–O–C stretching in the grafted hydroxy polyethylene glycol ether side chains, confirming the typical PCE structure [8]. Raman spectroscopy, a similarly widely used technique, assesses structural quality, defects, layer stacking, and polymer interactions via inelastic scattering of monochromatic light. In Xu et al.’s study [9] on water-soluble GnPs, the spectra showed the characteristic D (~1362 cm^−1^) and G (~1587 cm^−1^) bands, as well as peaks at ~2717 and ~2935 cm^−1^ from the 2D structure, confirming exfoliated graphene layers. In pulsed phase thermography (PPT), Fourier-transforming temperature–time data produces phase images (phase grams) that reveal inhomogeneities due to particle clustering [10].

Guardia et al. [11] demonstrated scalable production of pristine graphene in aqueous dispersions stabilized by non-ionic surfactants with dispersion quality and stability using UV–Vis spectroscopy, centrifugation, and microscopy. Although this represents a scalable route for large-scale applications, the required equipment (e.g., high-shear mixers or continuous-flow ultrasonic reactors) may not be widely available in every laboratory due to cost and complexity, highlighting the need for a simple method to characterize dispersions and evaluate their electrical properties. Finally, Dimou et al. [12] proposed a straightforward approach that allows dielectric measurement equipment to be used directly in the field, forming the foundation for the present investigation.

Ultrasonication energy is a critical factor in nanomaterial dispersion. A recent study on 2D nanomaterials assisted with graphene quantum dots, showed that no universal ultrasonication energy exists, as optimal conditions are system-dependent and must be tailored to each material [13]. Zheng et al. [14] proposed that few layer graphene nanofluids prepared at 20 % amplitude for 135 min present the highest stability. This is in agreement with the research of Chuah et al. [15], who indicated that GO dispersion in water is not strongly dependent on sonication intensity, as mild sonication provides sufficient energy to overcome weak van der Waals interactions. According to Jiang et al. [16], using a 50 mL sample volume, sonication at 60 % amplitude for 60 min was found to be sufficient for the dispersion of GnPs, with no additional benefits observed upon increasing the ultrasonication time. Unlike conventional approaches based solely on nominal sonication parameters (amplitude and duration), this study focuses on the effective sonication outcome, as assessed by electrochemical impedance spectroscopy (EIS) measurements.

Anionic surfactants enhance transport at low concentrations via electrostatic repulsion, while cationic surfactants improve mobility at higher concentrations due to surface charge modification, indicating that no single surfactant is universally optimal [17]. Ma et al. [18] showed that surfactant-functionalized rGO reduced the Hansen distance (Ra), indicating improved solvent compatibility, consistent with DLS measurements showing a decrease in particle size (e.g., 2257 → 294 nm in dichloromethane). Nazari et al. [19] optimized GnPs dispersion using UV–Vis, particle sizing, TEM/SEM, and zeta potential (DLVO theory), and correlated results with molecular dynamic simulations, identifying a 400 ppm (0.04% *w*/*v*) ammonium surfactant as optimal. Zhang et al. [20] proved that low RGO nanosheet contents (0.05 wt.% and 0.1 wt.%) are capable of achieving uniform dispersion within the epoxy matrix, using Raman and FTIR. Pineda et al. [21] have recently investigated how different surfactants affect the aqueous dispersion of Ti_3_C_2_T_x_MXene dispersions (2D metal-carbide sheets) in water using UV–Vis, DLS, and zeta potential and proved that ionic surfactants near their critical micelle concentration strongly enhance stability.

According to Al-Sughayer et al. [22] dispersion with 0.0025 wt.% GnPs and 0.1 wt.% PCE exhibited greater stability over 24 h according to UV–Vis. In this research, two concentrations of PCE (0.05 and 0.1 wt.%) were considered along with 0.0025, 0.01, 0.025 and 0.05 wt.% GnPs, laying the ground for future research in the optimization of dispersion parameters. While low-solid-content GnPs and GO dispersions have just been studied by Li et al. [6], high-solid-content systems remain largely unexplored. Increased particle aggregation reduced light transmittance, making UV–Vis and other standard characterization methods unsuitable. To perform the described protocol, they used FTIR/Raman, SEM/AFM, and DLS, which are costly and lab-bound methodologies, making simple dispersion-evaluation methods essential for in situ applications. In the present work, the challenge of determining the suitable ultrasonic energy required to achieve a homogeneous dispersion is addressed using a simple, fast, and cost-effective approach. In our previous study [23], the effects of uniformly dispersed GnPs and SP on the electrical and flexural properties of cement and lime pastes were evaluated, while acoustic emission was employed to monitor early damage and assess their electromechanical response, aiming at the development of a self-sensing nanocomposite. In the previous study, a constant ultrasonic energy of 65 kJ, selected based on literature data, was applied to all suspensions. Conversely, the present work investigates the electrical behaviour of dispersions prior to incorporation into nanocomposites, aiming to determine the optimum ultrasonic energy required for each suspension rather than adopting a single value for all suspensions. The novelty of this investigation lies in the systematic comparative analysis of GnPs aqueous dispersions over a wide range of SP and GnP concentrations. Unlike previous studies limited to narrow concentration ranges and complex techniques, a simple method is employed here, facilitating practical transfer, while varying ultrasonic energies are applied to assess different dielectric responses, providing a comprehensive understanding of dispersion behaviour. This study investigates the combined effect of GnPs and SP on the electrical properties of dispersions under varying ultrasonic energies, prior to their incorporation into cementitious matrices for enhanced sensing. A full matrix of dispersions with different concentrations of both components was examined. Ultrasonic energy was applied in all dispersions, and their electrical properties were measured with EIS and compared with optical microscopy images. Optimum concentrations that create a conductive network will be suggested to be ultrasonicated in a specific range of ultrasonic energy. To this end, the experimental results will be correlated with the equivalent circuit modelling results (ECMs) to augment reliability.

## 2. Experimental Procedures

Different concentrations of graphene nanoplatelets (GnPs) and a superplasticizer (SP) in aqueous suspensions were prepared, with the exact amounts of them and bottled water outlined in Table 1. All constituents were weighed, and the suspension was subjected to ultrasonication in a 150 mL beaker, which lies within the probe’s operational volume range (200 mL–1 L). The experimental setup is explicitly presented in [24].

### 2.1. Materials

The nanomaterial dispersions were prepared using commercially available materials, such as bottled water (Vikos S.A., Ioannina, Greece), a superplasticizer (SP) (ViscoCrete^®^ 5600 HS (Sika Hellas ABEE, Thessaloniki, Greece)), and graphene nanoplatelets (GnPs) of type N006-010-P (Angstron Materials Inc., Dayton, OH, USA). Aqueous suspensions were used as a simplified and controlled medium to isolate the effects of ultrasonic treatment during the initial dispersion stage, prior to cement hydration. The GnPs were added at concentrations of 0.15 wt.%, 0.50 wt.% and 1.00 wt.%, by weight of solids, with a water/binder ratio = 0.55. Five (5) different ratios of superplasticizer to nanomaterial concentration were examined (namely 0, 1, 2, 4 and 8) that hereafter will be named as SP0 series, SP1 series, SP2 series, SP4 series and SP8 series, respectively. The above ratios were determined in a previous article [23] of the research team, taking into account that the SP dosage must remain within the manufacturer’s recommended range (0.2–2.0 wt.% by weight of cement) as specified in the technical datasheet (Sika ViscoCrete^®^-5600 HS, 2019) [25]. The mix design presented in Table 1 was defined on a mass basis. The total mass of the solid binder was first determined, and the water content was adjusted according to the prescribed water-to-binder (w/b) ratio. The GnP content was then expressed as a weight percentage of the binder mass. Subsequently, the superplasticizer (SP) dosage was calculated based on a predefined SP-to-GnP mass ratio. This approach ensures a consistent and reproducible definition of all mixture components. Sample 4.00SP_0.50GnPs was included only for preliminary dispersion assessment and comparison purposes, and it was not used for the fabrication of the final nanocomposites [23] due to its SP content exceeding the recommended dosage range. Furthermore, a full set of mixtures without nanomaterials was employed as the SP reference, including 0.15SP_0.00GnPs, 0.30SP_0.00GnPs, 0.60SP_0.00GnPs and 1.20SP_0.00GnPs. The reference samples did not contain GnPs; however, their composition was determined based on the formulations corresponding to dispersions with 0.15 wt.% GnPs. For each reference sample, the SP dosage was selected to match the corresponding SP-to-GnP ratio (1:1, 2:1, 4:1, or 8:1), allowing for direct comparison with the respective GnP-containing dispersion.

GnPs maintain enhanced electronic conductivity and high aspect-ratio conductive pathways that make them effective in composite and electrochemical applications. They possess a high surface area, excellent electrical and thermal conductivity, mechanical strength, and the presence of oxygen-containing functional groups such as hydroxyl (–OH), carbonyl (C=O), and carboxyl (–COOH) on their edges or basal planes, which can enhance dispersion in solvents or polymer matrices and provide sites for chemical functionalization [22].

The conformation of SP molecules in dispersions is strongly influenced by the ionic environment. At low ionic strength, electrostatic repulsion between the negatively charged carboxylate groups (–COO^−^) leads to an extended polymer conformation. In contrast, at high ionic strength, the presence of counterions (e.g., Ca^2+^, Mg^2+^, Na^+^, and Cl^−^ included in Vikos water) screens these charges, promoting a coiled conformation of the polymer backbone. Nevertheless, the polyethylene glycol side chains remain extended in the aqueous medium, providing steric stabilization regardless of the backbone conformation [26].

### 2.2. Methods

Ultrasonication was performed using a VCX-500 ultrasonic processor equipped with a CV-334 probe (Sonics & Materials, Newtown, CT, USA). The ultrasonic system operated at a frequency of 20 kHz, with a maximum power output of 500 W and a probe tip diameter of 13 mm. Accordingly, amplitude values alone are not sufficient to fully describe the actual energy delivered to the nanofluid during sonication. High acoustic power can cause heating of the dispersion, so a pulsed operation (e.g., 15 s on and 30 s off) was applied, while the temperature was monitored throughout the process using an immersed thermometer. The temperature increased from approximately 26 °C to 50 °C due to ultrasonic heating throughout the duration of the experiment. The total sonication time was 4.5 h, corresponding to an energy input of 100 kJ. The reported ultrasonic energy input (~100 kJ) corresponds to the cumulative acoustic energy delivered during the effective sonication (ON) periods. Specifically, the process consisted of 360 cycles, each comprising 15 s of ultrasonication followed by 30 s of rest, resulting in a total active ultrasonication time of 5400 s (360 × 15 s). The energy input, *E*, was estimated using the following relationship:

*E* = *P*_eff_ ∗ *t*_ON_ (1)

where *P*_eff_ is the effective acoustic power transferred to the medium, and *t*_ON_ is the total active ultrasonication time. Under the experimental conditions described, this corresponds to an effective average power of approximately 18 W, yielding a total energy input of approximately 97.2 kJ (~100 kJ) (*E* = *P*_eff_ ∗ *t*_ON_ = 18 × 5400 = 97,200 J = 97.2 kJ).

Importantly, the ultrasonic power setting of the VCX-500 probe sonicator was kept constant throughout all experiments. Therefore, variation in energy input was achieved exclusively through adjustment of the total ultrasonication time (number of pulse cycles), rather than changes in amplitude or nominal power. Any interruptions (e.g., power loss or temporary stopping) did not reset the process, and the accumulated energy is considered additive over the total active ultrasonication time. For a comparison across samples, the energy input can be expressed as energy density (kJ/mL), which accounts for the dispersion volume and allows for normalization between experiments. This is particularly relevant in sonochemical processes where the cavitation field and energy distribution depend strongly on volume and geometry [27]. Regarding reproducibility, probe ultrasonication efficiency is highly sensitive to probe positioning, including the distance between the probe tip and the vial bottom, as well as lateral centring. To minimize this source of variability, all experiments were performed using identical 150 mL glass vials where the volume of water was set to 100 mL, with a fixed and reproducible probe immersion depth and geometry maintained throughout all runs of dispersions. This ensured consistent hydrodynamic and cavitation conditions across samples.

Electrochemical Impedance Spectroscopy (EIS) measurements were conducted during the ultrasonication process using a Dielectric Thermal Analysis System (DETA-SCOPE^®^, ADVISE, Chios, Greece). The system was coupled with an interdigital, film-shaped dielectric sensor (IDEX^®^, Netzsch^®^, Selb, Germany). This sensor was selected due to its compact geometry, with dimensions of approximately 10 mm in width and 30 mm in length, and since it provides high accuracy, it is suitable for the present application, as also reported by Dimou et al. [12]. Furthermore, the lightweight design of the impedance measurement device enhances the portability of the experimental setup, allowing for convenient use in a broad range of applications. The DETA-SCOPE^®^ applies a sinusoidal excitation voltage with an amplitude of 10 V over a frequency range of 0.1 Hz to 100 kHz. Each measurement is about 2.285 times larger than the previous one, showing exponential growth rather than a constant additive step. EIS measurements were conducted throughout the ultrasonic treatment up to 100 kJ, with data collected at intervals of 10 kJ. For each dispersion, 15–20 impedance measurements were recorded during ultrasonication. Since no significant variation in impedance was observed between closely spaced cycles, approximately five (5) representative cycles were selected based on the evolution of the impedance curves. The entire procedure was repeated three (3x) times for each dispersion to ensure reproducibility. The probe was immersed to approximately one-quarter (¼) of the liquid depth. All measurements were performed after completion of the ultrasonic treatment. Following sonication, the ultrasonic probe was removed from the suspension, and the sensor was immersed in the beaker containing the nanofluid for data acquisition.

The processing and interpretation of impedance data can be carried out through the analysis of an equivalent circuit model (ECM). However, this approach is often challenging, as multiple circuit configurations may provide equally good fits to the experimental data [28]. The issue of model non-uniqueness can be mitigated by a thorough understanding of the physicochemical processes and phases present in the system under investigation, such as double-layer capacitance, dispersion resistance and polarization resistance. Finally, to elucidate the electrochemical mechanisms, the dispersion was evaluated using optical microscopy with a Microscope VIS Kern (Balingen-Frommern, Germany) on samples ultrasonicated at selective ultrasonic energy levels. A small droplet of the suspension was collected using a pipette and placed between two glass slides (25 mm × 76 mm × 1 mm). The samples were examined immediately after preparation, without allowing the liquid to evaporate. Samples of all dispersions were investigated under different ultrasonic energies, and representative results are presented below, with emphasis on optimizing the ultrasonic energy. The observations were performed using 3.5× and 4× objective lenses (35× and 40× total magnification, respectively, with a 10× eyepiece), depending on the field of view required. The corresponding scale bars are provided in the micrographs. The goal was to determine the minimum energy required to achieve a homogeneous dispersion network.

## 3. Experimental Results

### 3.1. Optical Microscopy

The superplasticizer used (Sika ViscoCrete^®^ 5600 HS) belongs to the class of polycarboxylate ether (PCE)-based polymers. These materials typically consist of a poly-acrylic acid or poly-methacrylic acid backbone, [–CH_2_–CH(COO^−^)–]n, bearing negatively charged carboxylate groups, (–COO^−^) [29], onto which polyethylene oxide (PEO) side chains, –(O–CH_2_–CH_2_)_m_–OH, are grafted [8]. In polycarboxylate-based dispersants, the backbone provides adsorption and anionic groups (–COO^−^), while the side chains enable steric stabilization. In GnP dispersions, most ionic species originate from the aqueous medium and the dispersant. The –COO^−^ groups are covalently bound to the polymer, whereas Na^+^/K^+^ counterions are mobile in suspension and can be partially replaced by Ca^2+^ in cementitious environments, leading to stronger interactions and possible ionic bridging [30]. SP molecules cannot be observed by optical microscopy, nevertheless their effect with the mixing of GnP particles have been revealed.

**Figure 1:**
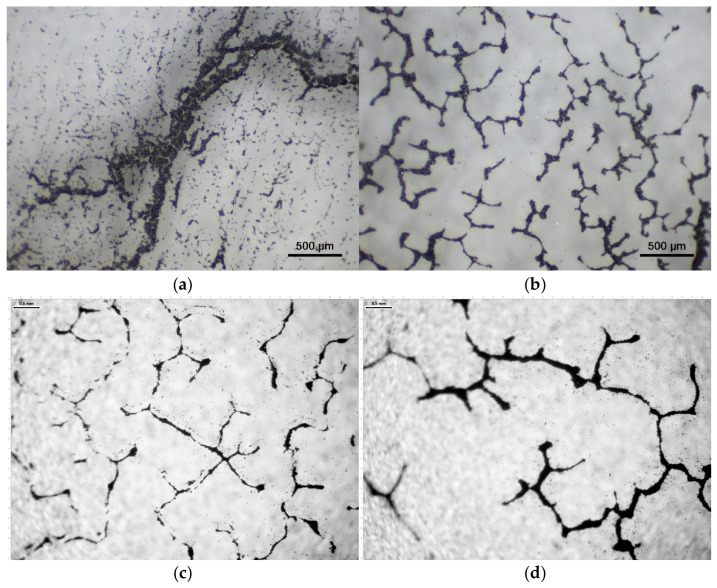
Optical microscopy image for the 0.00SP_1.00GnPs aqueous suspension at (**a**) 50 kJ and (**b**) 80 kJ, 1.00SP_1.00GnPs suspension at (**c**) 65 kJ, and 2.00SP_1.00GnPs at (**d**) 45 kJ. Scale bar is 0.5 mm for all images.

**Figure 2:**
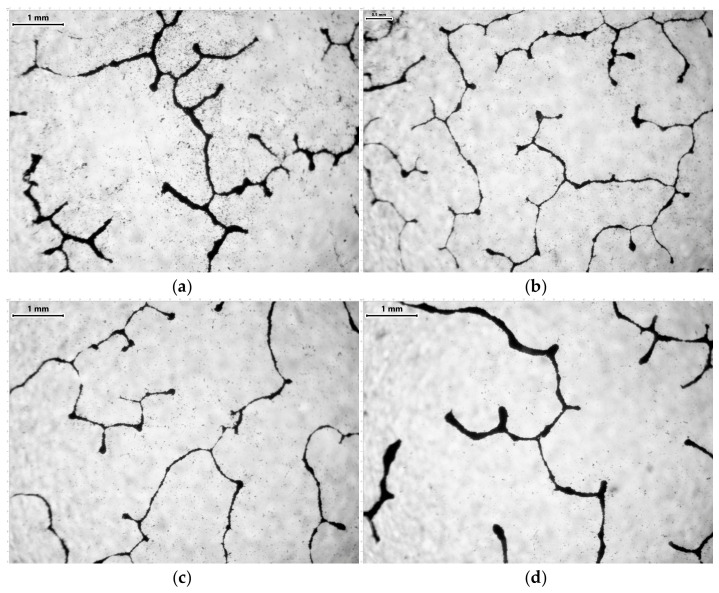
Optical microscopy image for the 1.00SP_0.50GnPs dispersion at (**a**) 30 kJ and (**b**) 100 kJ, (**c**) 2.00SP_0.50GnPs at 45 kJ, and (**d**) 4.00SP_0.50GnPs at 45 kJ. Scale bar is 1 mm for all images.

Optical microscopy observations (Figure 1 and Figure 2) reveal that regardless of the dispersion conditions, graphene nanoplatelets systematically form ramified, branched agglomerates rather than being fully individualized. However, the morphology of these agglomerates strongly depends on the dispersion conditions. Increasing both the superplasticizer (SP) content and the ultrasonic energy leads to finer and more extended branching structures, indicative of improved dispersion. In the 0.00SP_1.00GnPs dispersion agglomerates are clear at 50 kJ, and to achieve homogeneous dispersion 80 kJ is formed, as can be seen in Figure 1a,b. The conductive network in the 1.00SP_1.00GnPs dispersion is created at 65 kJ and in the 2.00SP_1.00GnPs dispersion at 45 kJ, as can be seen in Figure 1c and Figure 1d, respectively. These observations suggest that increasing SP content reduces the ultrasonic energy required to achieve a connected network, while also refining the structure of the agglomerates. Importantly, the persistence of branched agglomerates under all conditions indicates that the SP is not sufficient to fully overcome van der Waals interactions between graphene nanoplatelets. Rather than being detrimental, this partial aggregation can be beneficial, as it preserves interparticle contacts necessary for the formation of an electrically conductive percolated network. At the same time, improved dispersion (via higher SP dosages and/or ultrasonic energy) leads to a finer and more homogeneous network, which is favourable for percolation by increasing the number of effective conductive pathways. Figure 1a,b were acquired using a 4× objective lens (40× total magnification) to better visualize the agglomerates present in the suspension. The same magnification was used for both images, as they correspond to the same suspension. The remaining micrographs (Figure 2a–d) were acquired using a 3.5× objective lens (35× total magnification) to provide a larger field of view and facilitate the observation of the overall dispersion and distribution of GnPs within the suspension.

The addition of a double (SP2), fourfold (SP4) and eightfold (SP8) concentration of SP to 0.50 wt.% GnPs facilitates the formation of a conductive network, requiring only a small amount of ultrasonic energy. To be more specific, a percolated network is achieved in the 1.00SP_0.50GnPs dispersion at 30 kJ, in 2.00SP_0.50GnPs at 45 kJ, and in the 4.00SP_0.50GnPs dispersion at 45 kJ, shown in Figure 2a,c and Figure 2d, respectively.

In agreement with the previous observations, these systems also exhibit ramified agglomerates whose characteristic size and branching decrease as dispersion improves. Ultrasonic energy induces cavitation, and the collapse of microbubbles generates micro-jets and shockwaves that break particle agglomerates and improve dispersion [12]. Another remarkable result is that the application of ultrasonic energy as high as 100 kJ in the 1.00SP_0.50GnPs aqueous dispersion, shown in Figure 2b, appears to improve the dispersion state of GnPs. Microscopic observations indicate the formation of a more homogeneous network at higher sonication energy levels, suggesting improved dispersion. In some studies, similar energy inputs have been associated with re-agglomeration due to possible restacking of graphene-based nanomaterials [14]. However, no such behaviour was observed within the resolution limits of the present microscopy. This could be linked with the findings of Vikash et al., who reported that increasing shear rate (up to 1000 s^−1^) enhanced fine particle generation, while the Z-average particle size decreased from 250 nm to 65 nm. This correlation can only be made qualitatively, as ultrasonication induces localized extreme shear fields due to cavitation (10^6^–10^9^ s^−1^), which cannot be directly comparable to conventional shear flow conditions [31]. Nevertheless, a lower ultrasonic energy in the 1.00SP_0.50GnPs dispersion is preferred over 100 kJ to ensure energy efficiency while still achieving network formation.

### 3.2. EIS Results

**Figure 3:**
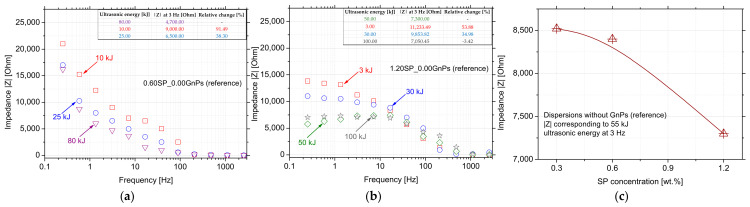
Bode plots in suspensions with varied concentrations of SP without GnP addition, (**a**) 0.60 wt.% and (**b**) 1.20 wt.%, and (**c**) impedance magnitude at 3 Hz vs. SP concentration corresponding to 55 kJ.

The Bode plot of reference dispersions (aqueous dispersions with varying concentrations of the SP) in Figure 3 presents the impedance spectrum for several values of applied ultrasonication energy. The impedance response is typically governed by a combination of capacitive interfacial processes and conductive percolation pathways. At higher frequencies, capacitive impedance decreases, reducing the overall impedance. The frequency range (0.1 Hz–10 kHz) probes interfacial polarization at low frequencies and conductive transport at high frequencies. For the interpretation of the Bode plot, intermediate frequencies (i.e., between 1 Hz and 1 kHz) are selected, away from polarization effects and parasitic contribution from cables and measurement circuits, where the response more reliably reflects the intrinsic properties of the material [32].

According to González-Aviña [33], at intermediate concentrations, the superplasticizer molecules may form transient associative structures, which could be associated with changes in viscosity and intermolecular interactions. These effects may increase resistance to flow and potentially reduce the efficiency of ultrasonic energy transfer, thereby requiring higher energy input, such as 80 kJ in the case of 0.60SP_0.00GnPs, as can be seen graphically in Figure 3a. At higher concentrations, a more homogeneous state may be observed, which may be associated with changes in the spatial distribution of polymer chains in the dispersion. This may influence the resulting microstructural response to ultrasonic energy input, potentially affecting the overall energy demand of the process, as shown in Figure 3b, for effective dispersion at 50 kJ in the case of the 1.20SP_0.00GnPs dispersion. Additionally, changes in cavitation behaviour under ultrasonic excitation may be associated with the observed trends [34]. However, a detailed analysis of bubble dynamics and cavitation efficiency is beyond the scope of the present study. Finally, the impedance at low frequency (3 Hz) decreases as the SP concentration increases, as can be noticed in Figure 3c. This is consistent with the fact that ionic conductivity increases with SP concentration.

The Bode plot alone cannot be used to conclusively identify the presence or absence of agglomeration in graphene-based dispersions. This limitation arises from the non-specific nature of the impedance magnitude (|Z|), as a decrease in |Z| may originate from multiple underlying mechanisms. Reduced impedance values may reflect improved dispersion and the breakdown of agglomerates, but they can also result from increased ionic conductivity of the suspension, variations in double-layer capacitance, or electrode-related effects. The Nyquist plot is often preferred over the Bode plot, since the imaginary impedance (Z″) plotted versus the real impedance (Z′) provides a direct visualization of the system’s resistive and capacitive elements. The high-frequency intercept (*R*_s_) reflects the ionic resistance of the aqueous medium. The observed semicircle in the present study is attributed to interfacial polarization effects and the formation of a percolative graphene nanoplatelet network, rather than a well-defined charge transfer resistance associated with Faradaic processes [35].

**Figure 4:**
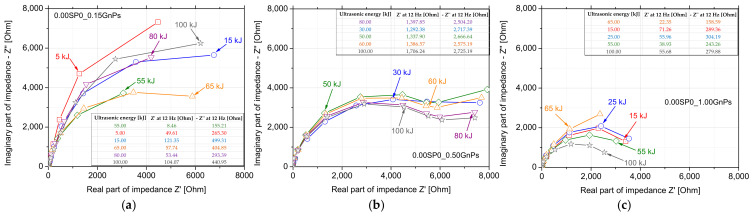
Nyquist plots in dispersions with (**a**) 0.15, (**b**) 0.50 and (**c**) 1.00 wt.% GnPs without SP (SP0 series).

**Figure 5:**
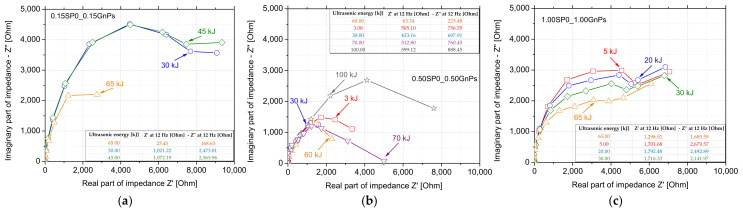
Nyquist plots in dispersions containing (**a**) 0.15, (**b**) 0.50, and (**c**) 1.00 wt.% GnPs, with the SP at equal weight fraction to the nanomaterial (SP1 series).

**Figure 6:**
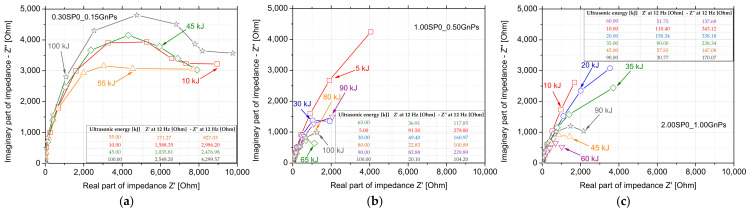
Nyquist plots in dispersions containing (**a**) 0.15, (**b**) 0.50, and (**c**) 1.00 wt.% GnPs, with the SP at double weight fraction to the nanomaterial (SP2 series).

**Figure 7:**
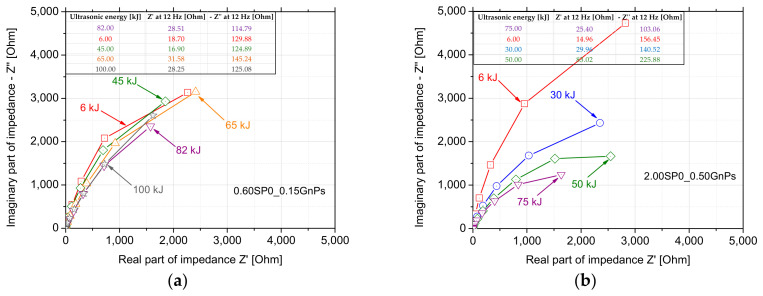
Nyquist plots of dispersions containing (**a**) 0.15 and (**b**) 0.50 wt.% GnPs, with the SP at fourfold weight fraction to the nanomaterial (SP4 series).

Representative cycles are shown for clarity. The impedance response shows a gradual evolution across successive cycles, while intermediate cycles exhibit a continuous transition and do not affect the overall trends. At low ultrasound energy, the Nyquist response typically exhibits a larger and often depressed semicircle, indicating interfacial heterogeneity that is likely related to the presence of graphene agglomerates in the dispersion. This trend is evident in 0.00SP_0.15GnPs at 15 kJ (Figure 4a); 1.00SP_1.00GnPs at 5, 20, and 30 kJ (Figure 5c); 0.30SP_0.15GnPs at 10 kJ (Figure 6a); and 2.00SP_0.50GnPs at 6 kJ (Figure 7b). At low frequencies, the Nyquist plot exhibits a linear region with an approximately 45° slope, characteristic of Warburg-type impedance, indicating diffusion-controlled processes within the system. This suggests diffusion limitations associated with ion transport in a heterogeneous and non-ideal structure, as Warburg-type behaviour appears in all spectra of 0.00SP_0.50GnPs regardless of ultrasonic energy (Figure 4b). Diffusion limitation is also apparent in 0.15SP_0.15GnPs at 30 and 45 kJ; 1.00SP_1.00GnPs at 5, 20, and 30 (Figure 5a,c); as well as in 0.30SP_0.15GnPs at 10 and 45 kJ (Figure 6a), indicating that the GnP dispersion in these systems may be insufficient to establish a continuous and highly conductive network.

In contrast, at higher ultrasound energy, the Nyquist plot shows a significant reduction in semicircle diameter, indicating lower effective suspension resistance, likely due to improved dispersion and partial breakdown of graphene agglomerates. This reflects enhanced charge transport across phase boundaries without involving Faradaic processes. The semicircle also becomes more defined and closer to ideal behaviour, as seen in 0.00SP_0.15GnPs at 65 kJ and 55 kJ spectra in Figure 4a, 0.15SP_0.15GnPs at 65 kJ spectra in Figure 5a and 0.30SP_0.15GnPs at 55 kJ in Figure 6a. The frequency of 12.41 Hz in the corner of the Nyquist plots, selected in the transition region between capacitive and resistive behaviour, was used to compare samples, where polarization effects start to diminish and conductive contributions become more pronounced. At 12.41 Hz, the shift of the Nyquist response toward the origin is consistent with lower resistance and improved dispersion at a specific ultrasonication energy, as seen for 0.50SP_0.50GnPs at 60 kJ in Figure 5b.

The imaginary component’s peak value of the curve that corresponds to 65 kJ in 0.15SP_0.15GnPs, shown in Figure 5a, drops in absolute value, approximately −77% compared to that in 0.00SP_0.15GnPs at the same sonication energy, which is shown in Figure 4a. As a result, the lower −Z″ response may indicate improved conductive connectivity within the dispersion.

**Figure 8:**
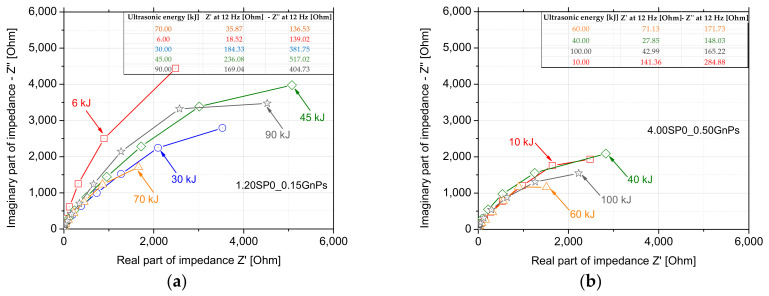
Nyquist plots of dispersions containing (**a**) 0.15 and (**b**) 0.50 wt.% GnPs with the SP at eightfold weight fraction to the nanomaterial (SP8 series).

In a Nyquist plot, the presence of a depressed semicircle is associated with interfacial polarization-related relaxation processes. The semicircle size is often related to the characteristic time scale of the process. A smaller semicircle generally suggests faster relaxation dynamics (shorter relaxation time), while a larger one is typically associated with slower dynamics. The system exhibits a relaxation process in the 2.00SP_0.50GnPs (Figure 7b) suspension at 50 kJ and in 4.00SP_0.50GnPs (Figure 8b) at the 60 kJ curve, specifically at 0.46 Hz. Relaxation features reflect interfacial polarization dynamics, and changes in their position or distribution should be interpreted comparatively across the curves corresponding to different ultrasonic energies, rather than as an absolute indicator of dispersion homogeneity.

## 4. Discussion of the Results

### 4.1. Randles Model

**Figure 9:**
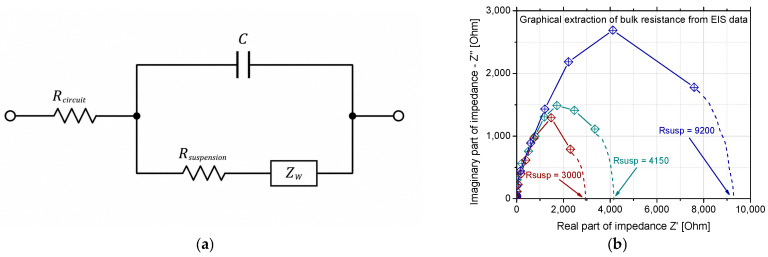
(**a**) Elements of the proposed Randles ECM and (**b**) graphical estimation of *R*_suspension_.

The Randles circuit is an equivalent electrochemical model of an electrode–electrolyte interface consisting of suspension resistance (*R*_s_), double-layer capacitance (*C*_dl_), charge-transfer resistance (*R*_ct_), and a Warburg element representing ion diffusion (Figure 9a). It is a simplified equivalent model and does not account for key operational factors in capacitive deionization systems, such as applied voltage, cell architecture, and ion concentration, all of which significantly influence the effective capacitance of the cell [36]. In the absence of Faradaic processes, the *R*_ct_ charge-transfer resistance term is not applicable and is replaced by a bulk/material resistance (*R*_suspension_ or *R*_susp_) associated with charge transport within the system. The suspension resistance corresponds to the overall ohmic contribution of the electrodes and external connections, but since in our case, it is mainly dominated by the contribution of the electrodes, contacts, and wiring, it will be referred as *R*_circuit_. Finally, *R*_suspension_ was extracted from the diameter of the semicircle in the Nyquist plot of Figure 9b, where applicable, and interpreted in terms of suspension conductivity processes. 

### 4.2. Analysis of R_suspension_

**Figure 10:**
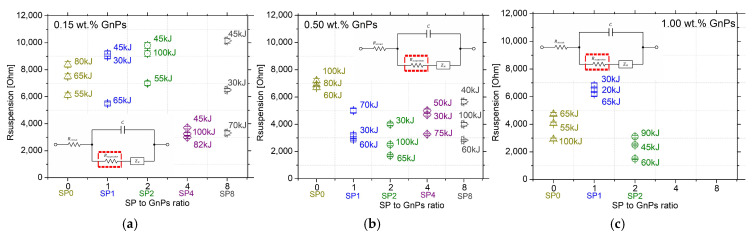
*R*_suspension_ variance extracted from Nyquist plots to SP/GnPs ratios reported as means ± standard deviation (*n* = 3 independent measurements) for (**a**) 0.15, (**b**) 0.50 and (**c**) 1.00 wt.% GnPs.

The selected optimum ultrasonic energy for 0.00SP_0.15GnPs seems to be 55 kJ, exhibiting the lowest *R*_susp_ value at 6000 Ohms in Figure 10a. Similarly, the optimum ultrasonic energy for 0.00SP_0.50GnPs seems to be 60 kJ and for 0.00SP_1.00GnPs seems to be 100 kJ, but according to optical microscopy (Figure 1b) 80 kJ is sufficient. For the SP1 series dispersions 60–65 kJ is sufficient to form a conductive network. Regarding the SP2 series, the selected optimum ultrasonic energy for 0.30SP_0.15GnPs seems to be 55 kJ. Similarly, the optimum ultrasonic energy for 1.00SP_0.50GnPs is 65 kJ, but according to optical microscopy (Figure 2a), 30–65 kJ is sufficient. For 2.00SP_1.00GnPs, 60 kJ exhibits the lowest *R*_susp_, but according to optical microscopy (Figure 1d), 45–60 kJ is suggested.

Excessive SP in the SP4 series may induce excessive interparticle separation, thereby reducing connectivity between nanoparticles, requiring higher ultrasonic energy to achieve homogeneous dispersion. This is why the optimum ultrasonic energy for 0.60SP_0.15GnPs is 82 kJ and for 2.00SP_0.50GnPs is 75 kJ. High ultrasonic energy values are also required for the SP8 series, 70 kJ for 1.20SP_0.15GnPs and 60 kJ for 4.00SP_0.50GnPs. According to microscopy at 0.50 wt.% GnPs and in high-SP-dosage suspensions (SP4 and SP8 series), 45 kJ ultrasonic energy was sufficient to achieve a conductive network (Figure 2c,d), with some distinct agglomerates though. This partial agglomeration may preserve interparticle contacts necessary for the formation of an electrically conductive percolated network, but for a safe material design the ultrasonic energy extracted from the Randles equivalent circuit is proposed.

## 5. Conclusions

This study examined the combined effect of graphene nanoplatelets (GnPs) and a superplasticizer (SP) on the electrical properties of aqueous dispersions under varying ultrasonic energies. Electrical behaviour was evaluated using electrochemical impedance spectroscopy (EIS) and correlated with optical microscopy observations. The experimental findings are supported by Randles equivalent circuit modelling, enhancing the reliability of the analysis.

○ The impedance at low frequency (3 Hz), corresponding to specific ultrasonic energy (55 kJ), decreases as the SP concentration increases in aqueous SP suspensions, denoting that ionic conductivity increases with the SP concentration.
○ According to microscopy, 45 kJ ultrasonic energy was sufficient to achieve a conductive network in 2.00SP_0.50GnPs and 4.00SP_0.50GnPs, with some distinct agglomerates though. Another remarkable result is that the application of ultrasonic energy as high as 100 kJ in the 1.00SP_0.50GnPs dispersion further enhanced GnP dispersion without affecting surface chemistry.
○ The optimum ultrasonic energy of 0.15, 0.50 and 1.00 wt.% GnP dispersions and varying SP-to-GnP ratios were identified though experiments and modelling:

- Ultrasonic energy of 55, 60 and 80 kJ accordingly for the 0.15, 0.50 and 1.00 wt.% GnP dispersions when the SP-to-GnP ratio is equal to 0;
- Ultrasonic energy of 65 kJ when the SP-to-GnP ratio is equal to 1;
- Ultrasonic energy of 55, 30–65 and 45–65 kJ when the SP-to-GnP ratio is equal to 2;
- Ultrasonic energy of 82 and 75 kJ for the 0.15, 0.50 wt.% GnP dispersions when the SP-to-GnP ratio is equal to 4;
- Ultrasonic energy of 70 and 60 kJ when the SP-to-GnP ratio is equal to 8.

## Figures and Tables

**Table 1:** Concentration of components of the GnP dispersions.

Code Name of the Suspension	SP Concentration (wt.%)	GnP Concentration (wt.%)	SP (g)	GnPs (g)	Water (mL)	Ratio SP/GnPs (-)
0.00SP_0.15GnPs	0.00	0.15	0.00	0.27	100	0
0.00SP_0.50GnPs	0.00	0.50	0.00	0.91	100	0
0.00SP_1.00GnPs	0.00	1.00	0.00	1.82	100	0
0.15SP_0.00GnPs (reference)	0.15	0.00	0.27	0.00	100	-
0.15SP_0.15GnPs	0.15	0.15	0.27	0.27	100	1
0.50SP_0.50GnPs	0.50	0.50	0.91	0.91	100	1
1.00SP_1.00GnPs	1.00	1.00	1.82	1.82	100	1
0.30SP_0.00GnPs (reference)	0.30	0.00	0.55	0.00	100	-
0.30SP_0.15GnPs	0.30	0.15	0.55	0.27	100	2
1.00SP_0.50GnPs	1.00	0.50	1.82	0.91	100	2
2.00SP_1.00GnPs	2.00	1.00	3.64	1.82	100	2
0.60SP_0.00GnPs (reference)	0.60	0.00	1.09	0.00	100	-
0.60SP_0.15GnPs	0.60	0.15	1.09	0.27	100	4
2.00SP_0.50GnPs	2.00	0.50	3.64	0.91	100	4
1.20SP_0.00GnPs (reference)	1.2	0.00	2.18	0.00	100	-
1.20SP_0.15GnPs	1.20	0.15	2.18	0.27	100	8
4.00SP_0.50GnPs	4.00	0.50	7.27	0.91	100	8

## Data Availability

The original contributions presented in this study are included in the article. Further inquiries can be directed to the corresponding author.
